# Night shift work and the risk of metabolic syndrome: Findings from an 8-year hospital cohort

**DOI:** 10.1371/journal.pone.0261349

**Published:** 2021-12-13

**Authors:** Wan-Ju Cheng, Chiu-Shong Liu, Kai-Chieh Hu, Yu-Fang Cheng, Kati Karhula, Mikko Härmä

**Affiliations:** 1 Department of Psychiatry, China Medical University Hospital, Taichung, Taiwan; 2 Department of Public Health, China Medical University, Taichung, Taiwan; 3 Center for Drug Abuse and Addiction, China Medical University Hospital, China Medical University, Taichung, Taiwan; 4 Department of Family Medicine, China Medical University Hospital, Taichung, Taiwan; 5 Management Office for Health Data, China Medical University Hospital, Taichung, Taiwan; 6 College of Medicine, China Medical University, Taichung, Taiwan; 7 Department of Endocrinology and Metabolism, Chanhua Christian Hospital, Chanhua, Taiwan; 8 Finnish Institute of Occupational Health, Helsinki, Finland; UAB School of Medicine, UNITED STATES

## Abstract

**Objectives:**

Studies concerning the risk of metabolic syndrome associated with night work have shown inconsistent findings, due to imprecise working time data and cross-sectional design. We used register-based daily working time data to examine the risk of incident metabolic syndrome associated with night shift work.

**Methods:**

Working time data collected between 2010 and 2018 of 5775 Taiwanese hospital workers were used to identify night shift workers and to calculate the number of night shifts. Metabolic syndrome was identified by annual occupational health examination results, which were linked to the working time data. Logistic regression models and generalized estimating equations were used to examine the association between night shift work and metabolic syndrome and the 5 components of metabolic syndrome.

**Results:**

Night shift work is associated with a higher risk of developing metabolic syndrome (adjusted OR = 1.36, 95% CI = 1.04 to 1.78) and high waist circumference (adjusted OR = 1.27, 95% CI = 1.07 to 1.78) compared to day work. Among night shift workers, increased number of night shifts was associated with high blood pressure (adjusted OR = 1.15, 95% CI = 1.01 to 1.31).

**Conclusions:**

Night shift work is associated with metabolic risk factors. Long-term effects of circadian rhythm disruption on metabolic disturbances needs to be further studied.

## Introduction

Metabolic syndrome, characterized by glucose intolerance, hypertension, dyslipidemia, and central obesity, increases the risks of cardiovascular disease, stroke, diabetes mellitus, and cognitive decline [1–4]. The prevalence of metabolic syndrome is between 5% and 20% among employees [5]. Metabolic syndrome can be diagnosed through occupational health examination, and interventions are cost-effective when conducted as a part of workplace health promotion to prevent cardiovascular events [6–8].

Scientific evidence for the association between shift work and metabolic syndrome is inconsistent [5, 9]. Canuto et al. concluded that evidence was not sufficient to support the association when sleep duration, behavioral, or sociodemographic characteristics were taken into account [5]. However, Wang et al. reported the increased risk of metabolic syndrome among workers who had been exposed to night shift work [9]. A meta-analysis of longitudinal studies found that shift work was associated with overweight and elevated blood glucose, whereas the association with lipid metabolism and blood pressure was not supported [10].

Among longitudinal studies concerning the association between shift work and metabolic syndrome, inconsistent definitions and measurements of working time probably constitute the main cause of inconsistent findings. Previous studies have used subjective data from questionnaires to categorize work schedules crudely into shift and non-shift workers [11–14], which ignored quickly changing work schedules among real-life shift workers [15]. Therefore, the real exposure of circadian disturbance due to night shifts was unknown. Furthermore, the definitions and cut-off values for shift work in previous studies have been either inconsistent or poorly described. For example, type of rotation and shift time (early morning/evening/night) may lead to different impact on health [16]. Recently it is recommended that working time characteristics replace subjective reports of shift work exposure in working time research [17], however, objective working time data is not always available to researchers.

In summary, while studies have confirmed the association between shift work and cardiovascular disease [18], studies concerning the risk of metabolic syndrome have provided inconsistent findings due to cross-sectional designs and a lack of high-quality exposure data. To fill up some of these research gaps, we used register-based daily working time data from a hospital employee cohort to examine the association of working night shifts with new-onset metabolic syndrome and its 5 components. In addition, we examined the association of night shift exposure with incident metabolic syndrome and its 5 components among night shift workers.

## Methods

### Participants and design

The participants of this study were 6607 hospital workers who underwent annual occupational health examinations from 2010 to 2018 in a medical center. All workers in service were mandated to undergo health examinations annually and the completion rate was 98.2%. The health examinations included blood tests, physical examinations, and illness history taking conducted by general physicians. Daily working time data of 365 days prior to each annual health examination were obtained from hospital-owned registry data. These data were retrieved from an electronic timecard system designed by the information technology department of the hospital, and the same system has been used for payroll.

**Fig 1:**
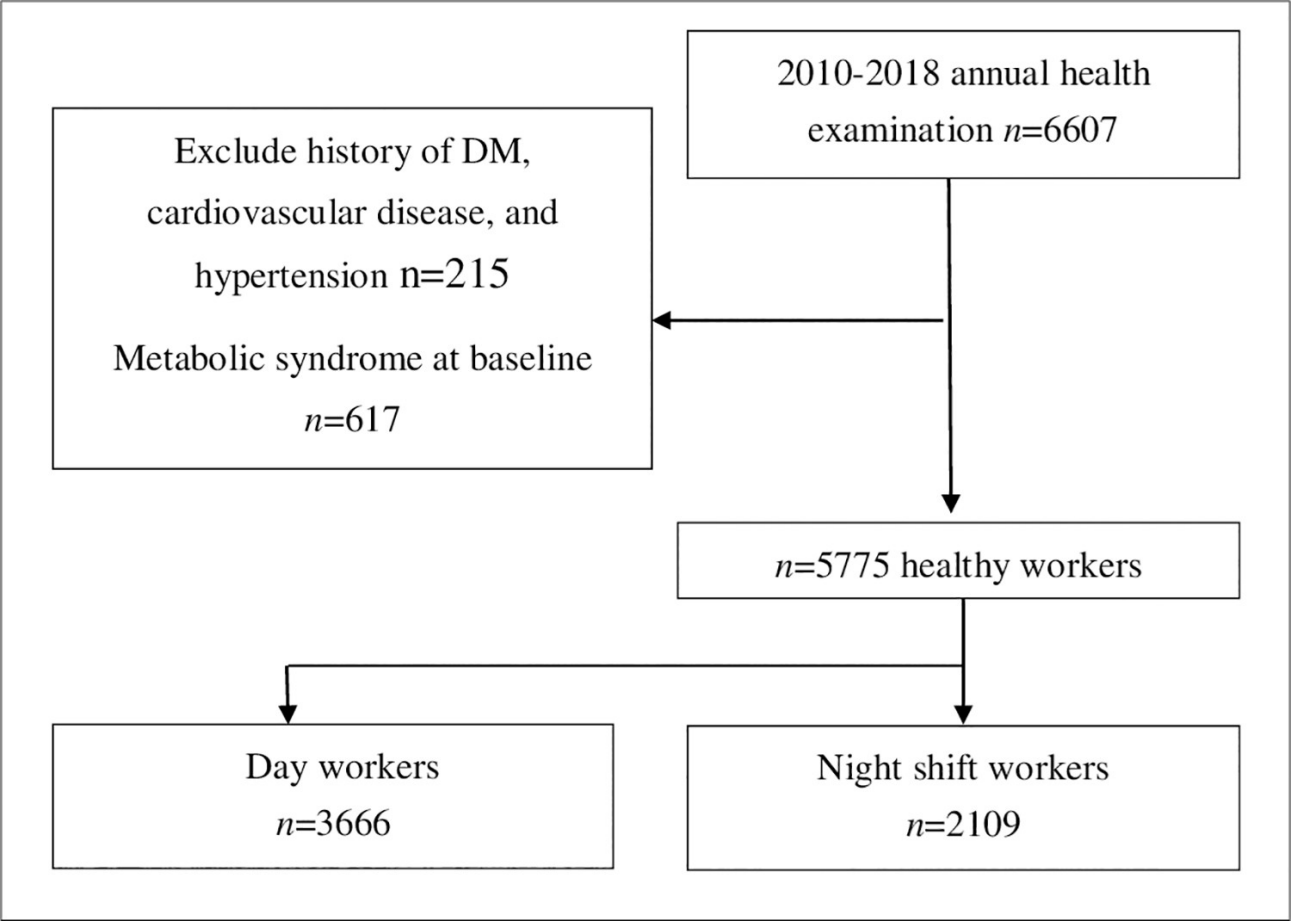
Flowchart of study participant selection from employees of a single medical center.

Participants were excluded if they had metabolic syndrome in the year of their first occupational health examinations (baseline) (N = 617) or if they had reported history of diabetes mellitus, hypertension, or cardiovascular disease (N = 215) (Fig 1). Finally, 5775 healthy workers were included in the final sample. The follow-up endpoint was defined as the year with new-onset metabolic syndrome, the last available record of health examination, or end of the study (i.e., 2018), whichever occurred first. The average follow-up period was 2.90 ± 1.63 years.

This study was approved by the Research Ethics Committee of China Medical University Hospital, Taiwan (CMUH106-REC3-152), and has conformed to the ethical norms and standards in the Declaration of Helsinki.

### Measures

The participants’ age and sex were obtained from the health examination data. Occupations were identified by employees’ identification number. Metabolic syndrome was confirmed by laboratory results of blood samples drawn following at least 8 hours of fasting. The definition of metabolic syndrome was the presence of three or more of the following criteria according to the guidelines of the National Heart, Lung, and Blood Institute and the American Heart Association [19]. (1) Fasting triglyceride ≥150mg/dL; (2) High-density lipoprotein (HDL) cholesterol: men <40mg/dL, women <50 mg/dL; (3) Fasting glucose: ≥100 mg/dL; (4) Waist circumference: men ≥90 cm, women ≥80 cm; (5) Systolic blood pressure ≥130 mmHg or diastolic blood pressure ≥85 mmHg. Baseline HDL cholesterol data was missing in 3839 participants, and they were considered positive in calculating.

Register-based working time characteristics of 365 days before each health examination were retrieved. Using the electronic timecard data, which tracks the starting and finishing working hours of each worker, we identified night shifts as ≥3 working hours between 0 AM and 5 AM according to the definition by International Agency for Research on Cancer [16]. We identified 3666 day workers who never worked night shifts during the study period. The other 2109 workers had averagely at least one night shift per month and were identified as night shift workers. We calculated the exact number of night shifts and weekly working hours of each year.

### Statistical analysis

We examined the differences in demographic characteristics, working time characteristics, metabolic syndrome incidence, and baseline metabolic components between day workers and night shift workers using Mann-Whitney U tests and chi-square tests.

The association between number of night shifts and metabolic syndrome was examined in two ways. First, we examined the association between night shift workers (reference group were day workers) and new-onset metabolic syndrome and 5 components using logistic regression analysis. In the crude models, we included age and sex as confounders. We further included occupation, weekly working hours, and baseline metabolic risk factors in the adjusted models. Second, in the night shift worker group, we examined the association between number of night shifts and new-onset metabolic syndrome and 5 components using general estimation equations (GEE). Those with missing values for working time or health examination results during the study period were assumed to have taken leaves of absence. Data after the year of absence were not included in the GEE analysis because their health conditions may have changed largely during the absence period. Because the study participants had different number of night shifts each year, we used yearly data for the predicting variable in the models. The distribution of number of night shifts was right-skewed and log-transformed. Age, sex, and working hours were included as covariates in the adjusted models (see statistical details in S1 File). All data were linked and analyzed using SAS software 9.4 (SAS Institute, Cary, NC, USA).

## Results

The occupations of the study participants were nurses (56.9%), medical technicians (22.2%), administrative clerks (16.2%), and pharmacists (4.8%). Physicians and outsourced workers were not included in this working time registry and were therefore not included in this study. Compared with day workers, night shift workers were younger (27.6 vs. 32.9 years old) and had a longer weekly working hours (38.7 vs. 36.8 h). The two groups had a similar incidence of metabolic syndrome (124 [5.9%] vs. 220 [6.0%], p = 0.85) over the follow-up period (Table 1). The incidence rates of metabolic syndrome in day workers and night shift workers were 21 and 20 cases per 1000 person-years, respectively. Day workers had a higher prevalence of elevated blood pressure than night shift workers at baseline. The incidence rates of high triglyceride, low HDL cholesterol, high glucose, high waist circumference, and high blood pressure among day workers were 19, 44, 24, 40, 31 cases per 1000 person-years, respectively. The incidence rates of the five components abnormalities among night shift workers were 12, 38, 17, 30, 21 cases per 1000 person-years, respectively.

**Table 1:** Demographic and working time characteristics of the study participants, with p-values for chi-square tests and Mann-Whitney U tests comparing between day and night shift workers (N = 5775).

	Day workers (*n* = 3666)		Night shift workers (*n* = 2109)		*P* value
Age (mean and SD)	32.9	(8.7)	27.6	(5.1)	<0.01
Sex (female) (N and %)	3152	(86.0)	1951	(92.5)	<0.01
Occupations (missing = 27)					<0.01
Nurses	1508	(41.4)	1762	(83.7)	
Medical technicians	1045	(28.7)	231	(11.0)	
Administrative clerks	858	(23.6)	71	(3.4)	
Pharmacists	232	(6.4)	41	(2.0)	
Working hours (week^-1^) (mean and SD)	36.8	(3.7)	38.7	(1.9)	<0.01
Number of night shifts (year^-1^) (mean and SD)	1.0	(2.5)	54.6	(38.2)	<0.01
Baseline metabolic abnormalities (N and %)					
High triglyceride	234	(6.4)	108	(5.1)	0.05
Low HDL cholesterol (missing = 3839)	198	(14.5)	76	(13.3)	0.49
High glucose	257	(7.0)	123	(5.8)	0.08
High waist circumference	548	(15.0)	340	(16.1)	0.24
High blood pressure	467	(12.7)	204	(9.7)	<0.01
New-onset metabolic syndrome (N and %)	220	(6.0)	124	(5.9)	0.85

Table 2 shows that in the logistic models, night shift workers had an elevated risk of new-onset metabolic syndrome (adjusted odds ratio [aOR] = 1.36, 95% confidence interval [CI] = 1.04–1.78, p = 0.03), and high waist circumference (aOR = 1.27, 95% CI = 1.07–1.51, p = 0.01). We further added triglyceride, glucose, waist circumference, and systolic blood pressure at baseline in the models, and found that night shift work remained associated with metabolic syndrome (aOR = 1.40, 95% CI = 1.06–1.85, p = 0.02) and high waist circumference (aOR = 1.44, 95% CI = 1.17–1.78, p < 0.01).

**Table 2:** Odds ratio (OR) and 95% confidence interval (CI) of night shift work (reference: Day work) for new-onset metabolic syndrome and the five components in logistic regression models (n = 5775).

Outcome variables	Model 1		Model 2		Model 3	
	OR (95% CI)	*P*	OR (95% CI)	*P*	OR (95% CI)	*P*
Metabolic syndrome	**1.47 (1.14, 1.88)**	**<0.01**	**1.36 (1.04, 1.78)**	**0.03**	**1.40 (1.06, 1.85)**	**0.02**
High triglyceride	1.18 (0.92, 1.52)	0.20	1.17 (0.89, 1.54)	0.27	1.22 (0.90, 1.63)	0.20
Low HDL cholesterol	1.04 (0.93, 1.17)	0.49	1.07 (0.94, 1.21)	0.31	1.09 (0.90, 1.32)	0.39
High glucose	1.27 (0.99, 1.61)	0.06	1.09 (0.84, 1.41)	0.53	1.06 (0.81, 1.40)	0.67
High waist circumference	**1.26 (1.07, 1.47)**	**<0.01**	**1.27 (1.07, 1.51)**	**0.01**	**1.44 (1.17, 1.78)**	**<0.01**
High blood pressure	1.07 (0.88, 1.30)	0.50	1.13 (0.92, 1.39)	0.26	1.22 (0.96, 1.55)	0.11

Model 1: adjusted for age and sex.

Model 2: adjusted for age and sex, occupation, and working hours.

Model 3: adjusted for age, sex, occupation, working hours, and baseline metabolic risk factors.

Among night shift workers, number of night shifts was not associated with new-onset metabolic syndrome in both crude and adjusted models (Table 3). The results for each of the five components of metabolic syndrome showed that number of night shifts was significantly associated with high blood pressure (aOR = 1.15, 95% CI = 1.01–1.31, *P* = 0.04).

**Table 3:** Odds ratio (OR) and 95% confidence interval (CI) of number of night shifts for metabolic syndrome and the five components in generalized estimating equations among night shift workers (N = 2109).

	Model 1		Model 2	
Outcome variables	OR (95% CI)	*P*	OR (95% CI)	*P*
Metabolic syndrome	0.93 (0.78, 1.11)	0.44	0.96 (0.79, 1.15)	0.64
High triglyceride	0.88 (0.77, 1.01)	0.07	0.93 (0.81, 1.07)	0.29
Low HDL-C	0.97 (0.89, 1.06)	0.54	0.98 (0.90, 1.08)	0.69
High glucose	0.99 (0.87, 1.13)	0.91	1.02 (0.89, 1.16)	0.82
High waist circumference	0.99 (0.93, 1.06)	0.85	1.02 (0.95, 1.09)	0.65
**High blood pressure**	**1.15 (1.01, 1.30)**	**0.04**	**1.15 (1.01, 1.31)**	**0.04**

Model 1: adjusted for age and sex.

Model 2: adjusted for age, sex, and working hours.

## Discussion

Using a longitudinal design and objective exposure data, we observed an elevated risk of developing metabolic syndrome and high waist circumference in night shift workers compared to day workers. Among night shift workers, number of night shifts was associated with high blood pressure.

This study is one of the few that has used register-based daily working time data to examine the risk of metabolic risk factors among shift workers. Earlier studies which showed increased risk of metabolic syndrome among night shift workers mostly used self-reported work schedule [12–14]. The association between night shift work and metabolic syndrome may be explained by disturbed sleep, adverse lifestyles, and psychosocial stress [20]. Earlier studies have suggested that an elevated risk of metabolic syndrome is more common in older workers and workers with over 10 years of shift work experience [11, 21]. Nevertheless, relative to participants in previous studies, our study participants were younger and may have fewer years of previous shift work experience. Our results showed that metabolic syndrome may develop within a short night shift work duration.

We also observed an association between night shift work and high waist circumference. At baseline, night shift workers had a slightly higher prevalence of high waist circumference than day workers, although statistically insignificant. Among the components of metabolic syndrome, high waist circumference has most commonly been reported to associate with night shift work [12, 22–24], followed by low HDL cholesterol and high fasting glucose. The mechanism of increased waist circumference among night workers is probably circadian rhythm misalignment [25, 26]. Shift workers showed diurnal cortisol pattern changes compared to day workers [27, 28], which may in turn leads to central obesity [29, 30].

In the night shift worker cohort, we found that a higher number of night shifts is associated with increased risk for high blood pressure. A review study observed that blood pressure measured during shift work is elevated compared to blood pressure during sleep period [31], showing the acute effects of shift work on blood pressure. However, few studies have examined the long-term impact of shift work on blood pressure. A meta-analysis showed that permanent night shift work is associated with more elevated blood pressure than rotating shift work [32]. The mechanism behind the association between night work and elevated blood pressure has been suggested to be circadian rhythm disruption [33] and sleep disturbance [34]. Night shift workers who work a high percentage of night shifts may keep a desynchronized circadian rhythm even during their off days, and thus exposed to more severe circadian rhythm disruption.

We did not find association between number of night shifts and metabolic syndrome. The association between night shift work and metabolic syndrome we observed in logistic regression models may be explained by lifestyle characteristics of night shift workers, such as diet and physical activities, rather than night shift burden. Nevertheless, blood pressure varies quickly with acute stress, we may find associations between night work and other metabolic abnormalities with longer follow-up time. Hypertension is a major modifiable cause of cardiovascular diseases [35], and to decrease blood pressure in patients with hypertension significantly reduces mortality [36]. Our findings that a higher number of shift work is associated with elevated blood pressure partially explained the increased cardiovascular risk among long-term shift workers [37]. We suggest that ambulatory blood pressure monitoring should be considered for shift workers with high cardiovascular disease risks.

The strength of this study is that we used continuous working time registry data and objective health examination data to explore the relationship between night work and metabolic syndrome. Findings for the dose–response effect of shift work on metabolic syndrome have been inconsistent [11, 24, 38] when the cumulative years of shift work were retrospectively collected by questionnaires and were therefore subject to recall bias and healthy worker effects. For example, longitudinal studies have revealed that workers with obesity or depressive symptoms tended to change from night shift to day work [15, 39]. The finding that former shift workers, but not current shift workers, had a higher prevalence of metabolic syndrome also supported the presence of the healthy worker effect among shift workers [40]. We have minimized the healthy worker effect by following a young night shift worker cohort with objective working time data. Nevertheless, shift workers who developed metabolic abnormalities or other acute adverse health consequences may have the tendency to decrease number of night shifts or change their work schedule to day work. In such case, we may have underestimated the association between night shift work and metabolic syndrome.

This study has some limitations. First, the follow-up period was rather short, partially because workers changed their jobs frequently and we were unable to access their working time and health data when they worked at other medical facilities. While elevated blood pressure developed in a short period in our study, other metabolic abnormalities may develop with a longer period of night shift exposure. In addition, history of shift work experience among the study participants was not collected in this study. The association between number of night shifts and metabolic risks may have been overestimated if the participants had a long history of night shift work experience before the study period. A nation-wide database regarding working time and health would provide a longer follow-up period across jobs and enable an examination of the health effect of long-term night work exposure. Second, the study sample was limited to healthcare workers. Healthcare workers usually have healthier lifestyles than workers in many other occupational sectors. However, the similar lifestyle among hospital workers may have reduced unobserved confounders in the statistical models. Third, data regarding baseline health behaviors and other vital variables including chronotype, physical activity, diet, alcohol drinking, smoking, work psychosocial conditions, and family burden were not available in this study. To include these variables in future studies would help clarify the mechanism of metabolic risks among night shift workers.

In conclusion, this study used continuous register-based working time data and health examination data in a hospital cohort. We found that night shift work was associated with metabolic syndrome and central obesity, and a higher night shift was associated high blood pressure in night shift workers. We suggest monitoring waist circumference and blood pressure in shift workers to detect of health risks and prevent further metabolic problems and cardiovascular disorders. In addition, future studies are needed to explore the mechanisms underlying the relationship between long-term circadian misalignment and metabolic risks.

## Supporting information

S1 File Statistical details for the general estimating equations. (DOCX)
