# Supplementary material for: Night shift work and the risk of metabolic syndrome: Findings from an 8-year hospital cohort
Source: PLoS One. 2021 Dec 13;16(12):e0261349. doi: 10.1371/journal.pone.0261349 (PMC8668137; doi:10.1371/journal.pone.0261349)
Supplement: S1 File — (DOCX) [file pone.0261349.s001.docx]

**S1 File**

In the general estimating equations estimating odds ratio of number of night shifts for metabolic syndrome, the mathematical formulation is:

$$\mathrm{logit}\left( y_{ij} \right)=\beta_{0}+\beta_{1}{x_{1}}_{ij}+\beta_{2}{x_{2}}_{i}+\beta_{3}\left[ log\left( {x_{3}}_{ij} \right) \right]^{2}+{CORR}_{exch}+e_{i}$$

$y_{ij}$ is 1 if participant i suffered from metabolic syndrome at time j

${x_{1}}_{ij}$ is age for participant i at time j

${x_{2}}_{ij}$ is gender for participant i

${x_{3}}_{ij}$ is cumulative number of night shift work for the past 1 year for participant i at time j

${CORR}_{exch}$ is exchangeable working correlation matrix

$e_{i}$ is random error term for participant i

SAS codes of the fully adjusted general estimating equations using SAS software 9.4 are as follows:

Proc genmod data=x descending;

class id sex;

model outcome=age sex workhour nightshift/dist=bin;

repeated subject=id/type=exch covb corrw;

run;
